# Diarylalkanoids as Potent Tyrosinase Inhibitors from the Stems of *Semecarpus caudata*

**DOI:** 10.1155/2021/8872920

**Published:** 2021-01-04

**Authors:** Phu H. Dang, Tho H. Le, Truong N. V. Do, Hai X. Nguyen, Mai T. T. Nguyen, Nhan T. Nguyen

**Affiliations:** ^1^Faculty of Chemistry, University of Science, 227 Nguyen Van Cu Street, Ward 4, District 5, Ho Chi Minh City, Vietnam; ^2^Vietnam National University, Quarter 6, Linh Trung Ward, Thu Duc District, Ho Chi Minh City, Vietnam; ^3^Cancer Research Laboratory, University of Science, 227 Nguyen Van Cu Street, District 5, Ho Chi Minh City, Vietnam

## Abstract

From a CHCl_3_-soluble extract of the stems of *Semecarpus caudata* (Anacardiaceae), two new diarylalkanoids, semedienone (**1**) and semetrienone (**2**), were isolated. Their structures were elucidated based on NMR spectroscopic data interpretation. These compounds possess strong tyrosinase inhibitory activity with the IC_50_ values of 0.033 and 0.11 *μ*M, respectively. Docking studies of **1** and **2** with *oxy*-tyrosinase were carried out to analyze their interactions. Accordingly, semedienone (**1**) showed good interactions with the peroxide group and amino acid residues. The biosynthesis of the isolated diarylalkanoids was proposed.

## 1. Introduction

Melanin is a pigment that is essential for protecting human skin against UV radiation. However, the abnormal accumulation of melanin induced skin pigmentation disorders. Melanogenesis is a complex process to produce melanin under control of tyrosinase. Tyrosinase (EC 1.14.18.1) is a binuclear copper-containing monooxygenase, which catalyzes the oxidation of phenol to the corresponding *o*-quinone [1,2]. Tyrosinase is the main factor causing some dermatological diseases including freckles, age spots, and melasma. Hydroquinone, arbutin, kojic acid, azelaic acid, L-ascorbic acid, ellagic acid, and tranexamic acid are commercial tyrosinase inhibitors, which have been used as skin-whitening agents, but these compounds have certain drawbacks [3]. Thus, the finding of new efficient and safe antityrosinase agents is necessary for anti-hyperpigmentation drug development.

A previous study on the chemical constituents of *Semecarpus caudata* (Anacardiaceae), collected at Dong Nai Province in Vietnam, led to the isolation of six flavonoid derivatives and the evaluation of their tyrosinase inhibitory activity [4]. Our continued phytochemical study on the stems of *S. caudata* was carried out, leading to the isolation of seven compounds (**1**–**7**) including two new diarylalkanoids named semedienone (**1**) and semetrienone (**2**). These compounds were found to possess tyrosinase inhibitory activity. Semedienone (**1**) showed a strong effect with an IC_50_ value of 0.033 *μ*M, which makes it 1300 times more potent than that of kojic acid (IC_50_, 44.6 *μ*M). In addition, molecular docking studies of **1** and **2** with the *oxy*-form of the copper-bound *Streptomyces castaneoglobisporus* tyrosinase were performed.

## 2. Materials and Methods

### 2.1. General Experimental Procedures

Optical values were measured on a Shimadzu UV-1800 spectrophotometer (Shimadzu Pte., Ltd., Singapore). IR spectra were measured with a Shimadzu IR-408 infrared spectrometer (Shimadzu Pte., Ltd., Singapore). NMR spectra were acquired on a Bruker Avance III 500 spectrometer (Bruker BioSpin AG, Bangkok, Thailand). Chemical shifts are expressed as *δ* values. HRESIMS data were acquired on Bruker micrOTOF-QII mass spectrometer (Bruker Singapore Pte., Ltd., Singapore). Column chromatography was carried out using silica gel 60, 0.06–0.2 mm (Scharlau, Barcelona, Spain) and LiChroprep RP-18, 40−63 *μ*m (Merck KGaA, Darmstadt, Germany). Kieselgel 60 F_254_ or RP-18 F_254_ plates for TLC were purchased from Merck (Merck KGaA, Darmstadt, Germany). Tyrosinase (EC 1.14.18.1) from mushroom (3933 U·mL^−1^) and L-dihydroxyphenylalanine (l-DOPA) were obtained from Sigma-Aldrich (Sigma-Aldrich Pte., Ltd., Singapore). Other chemicals were of the highest grade available.

### 2.2. Plant Material

The stems of *Semecarpus caudata* were collected in the Ma Da Forest, Dong Nai Culture and Nature Reserve, Dong Nai Province, Vietnam, in April 2014. The plant was identified by Assoc. Prof. Dr. Hop Tran, Institute of Tropical Biology, Ho Chi Minh City, Vietnam. A voucher sample (MCE0002) has been deposited at the Division of Medicinal Chemistry, Faculty of Chemistry, University of Science, Ho Chi Minh City, Vietnam.

### 2.3. Extraction and Isolation

The dried powdered stems of *S. caudata* (7.0 kg) were exhaustively extracted in a Soxhlet extractor with MeOH (20 L, 3 h × 3) to yield MeOH-soluble extract (700 g). This extract was suspended in H_2_O (5 L) and successively partitioned with *n*-hexane (2 L) and CHCl_3_ (3 L) to give *n*-hexane (37 g)- and CHCl_3_ (500 g)-soluble fractions. The CHCl_3_-soluble fraction was chromatographed by silica gel column chromatography (15 × 150 cm) and eluted with EtOAc-*n*-hexane (0 : 100 ⟶ 100 : 0) and MeOH-CHCl_3_ (0 : 100 ⟶ 20 : 80) to afford 12 fractions (Fr.1−Fr.12). Fraction Fr.3 (4.5 g) was subjected to further silica gel column chromatography and was eluted with EtOAc-*n*-hexane (0–100%) to yield 4 subfractions (Fr.3.1−Fr.3.4). Subfractions Fr.3.2 (1.1 g) and Fr.3.3 (540 mg) were chromatographed over a silica gel column with EtOAc-*n*-hexane (0–100%) and purified by preparative TLC with EtOAc-*n*-hexane (20 : 80) and EtOAc-CHCl_3_ (10 : 90) to afford **5** (5.0 mg) and **7** (6.0 mg), respectively. Fraction Fr.5 (5.2 g) was separated by silica gel column chromatography with EtOAc-*n*-hexane (0–100%) and MeOH-CHCl_3_ (0–20%) to yield 5 subfractions (Fr.5.1−Fr.5.5). Subfraction Fr.5.3 (850 mg) was subjected to further silica gel column chromatography, eluted with Me_2_CO-CHCl_3_ (0–80%) to give **4** (5.0 mg). Fraction Fr.6 (0.9 g) was separated by normal-phase chromatography with EtOAc-*n*-hexane (0 : 100 ⟶ 80 : 20) and MeOH-CHCl_3_ (0 : 100 ⟶ 5 : 95) and reversed phase chromatography with H_2_O-MeOH (0–100%) and then purified by preparative TLC with AcOH-EtOAc-PhMe (4 : 16 : 80) to obtain **3** (4.0 mg) and **6** (4.0 mg). Fraction Fr.8 (4.5 g) was loaded onto a silica gel column and eluted with CHCl_3_-Me_2_CO (0–80%) and CHCl_3_-MeOH (0–20%) to yield 5 subfractions (Fr.8.1–Fr.8.5). Subfraction Fr.8.2 (630 mg) was further purified using a silica gel column with EtOAc-CHCl_3_ (0–80%) and preparative TLC with MeOH-CHCl_3_ (5 : 95) to give **1** (2.0 mg) and **2** (2.0 mg).

#### 2.3.1. Semedienone (**1**)

Yellow, amorphous solid; IR *v*_max_ (CHCl_3_): 3455, 1620, 1485, 1250, 1091 cm^−1^; ^1^H and ^13^C NMR (500 MHz, acetone-*d*_6_, see Table 1); HRESIMS *m/z* 321.0752 [M + Na]^+^ (calcd. for C_17_H_14_O_5_Na, 321.0739).

#### 2.3.2. Semetrienone (**2**)

Yellow, amorphous solid; IR *v*_max_ (CHCl_3_): 3305, 1650, 1430, 1245, 1085 cm^−1^; ^1^H and ^13^C NMR (500 MHz, acetone-*d*_6_, see Table 1); HRESIMS *m/z* 347.0902 [M + Na]^+^ (calcd. for C_19_H_16_O_5_Na, 347.0895).

### 2.4. Synthesis of 2,4,2′,4′-Tetrahydroxychalcone (**8**)

2,4-Dihydroxybenzaldehyde (276.3 mg, 2.0 mmol) and 2ʹ,4ʹ-dihydroxyacetophenone (152.1 mg, 1.0 mmol) were dissolved in 1 mL·H_2_O, and then 1 mL KOH 14 M was added. The resulting mixture was kept in the ultrasonic water bath at 80°C for 8 h. This reaction was monitored by TLC using MeOH-CHCl_3_ (1 : 9) mixture. After completion, the reaction mixture was quenched by acidification with HCl 3 M to pH ∼5 and cooled to 0°C to precipitate crude product, which was recrystallized with MeOH-H_2_O (1 : 3) mixture to afford pure chalcone. It was identified as 2,4,2′,4′-tetrahydroxychalcone (**8**), by comparison with authentic sample.

### 2.5. Tyrosinase Inhibitory Assay

All pure compounds were dissolved in DMSO and tested at concentrations ranging from 0.01 to 100 *μ*M. Assay mixtures in 0.1 M phosphate buffer pH 6.8 were prepared immediately before use, consisting of 100 *μ*L of tyrosinase solution (15 U/mL) and 1900 *μ*L of test solution. These mixtures were preincubated at room temperature for 30 min, followed by addition of 1000 *μ*L of l-DOPA 1.5 mM in pH 6.8 phosphate buffer and incubated at room temperature for 7 min. The absorbances (*A*) at 475 nm were acquired on Shimadzu UV-1800 spectrophotometer. The inhibitory percentage (*I*%) was calculated according to the formula: *I*% = ((*A*_control_ − *A*_sample_)/*A*_control_) × 100%. Data were represented as means ± standard error (*n* = 3). The IC_50_ values were determined by using GraphPad Prism software with multivariate nonlinear regression and *R*^2^ > 0.9. Kojic acid was used as positive control.

### 2.6. Molecular Docking

Docking studies of **1**, **2**, **8**, and the positive reference (kojic acid) were performed with Molecular Operating Environment 2016 (MOE 2016.0802) suite. The structures of these compounds were constructed by using the Builder module. Subsequently, all compounds were minimized up to 0.0001 gradients using the Amber12 : EHT force field. The crystal structure of the *oxy*-tyrosinase was taken from the Protein Data Bank (PDB code 1WX2). The caddie protein (ORF378) and water molecules were removed. The enzyme structure was prepared using the QuickPrep module. The binding site was determined based on the PLB (Propensity for Ligand Binding) score in the Site Finder module. The molecular docking was performed by Dock module, using Triangle Matcher placement, Induced Fit refinement, London dG, and GBVI/WSA dG scoring methods. Five top poses showed up based on the negative binding free energy value (*S* value). The best pose was selected to analyze the receptor-ligand interactions by using BIOVIA Discovery Studio Visualizer 2016.

## 3. Results and Discussion

### 3.1. Extraction and Isolation

**Figure 1:**
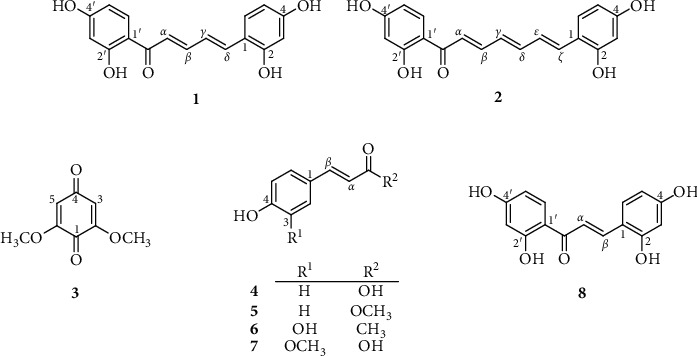
Structures of compounds **1**–**8**.

The dried powdered stems of *S. caudata* were exhaustively extracted in a Soxhlet extractor with MeOH to yield MeOH-soluble extract (700 g). This extract was successively partitioned to give the *n*-hexane (37 g)- and CHCl_3_ (500 g)-soluble fractions. The CHCl_3_-soluble extract of stems of *S. caudata* was repeatedly chromatographed using silica gel CC and preparative TLC to obtain seven compounds including two new diarylalkanoids named semedienone (**1**) and semetrienone (**2**). The known compounds were identified as 2,6-dimethoxybenzoquinone (**3**) [5], *p*-coumaric acid (**4**) [6], methyl *p*-coumarate (**5**) [7], *trans*-4-(3,4-dihydroxyphenyl)but-3-en-2-one (**6**) [8], and ferulic acid (**7**) [9] (Figure 1).

### 3.2. Structural Elucidation of Two New Isolated Compounds from *S. caudata*

**Figure 2:**
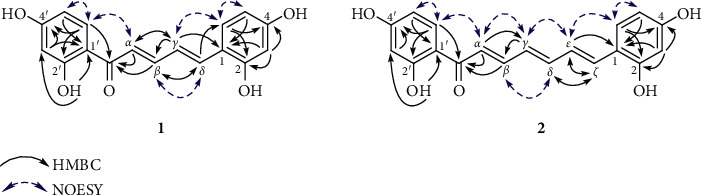
Significant HMBC (solid arrows) and selected NOESY correlations (blue dashed arrows) observed for **1** and **2**.

Compound **1** showed a molecular formula to be C_17_H_14_O_5_ based on the HRESIMS ion at *m/z* 321.0752 [M + Na]^+^ (calcd. for C_17_H_14_O_5_Na, 321.0739). The IR spectrum exhibited the presence of hydroxy (3455 cm^−1^) and carbonyl (1620 cm^−1^) functionalities. The ^1^H NMR spectrum showed signals for two 1,2,4-trisubstituted aromatic rings (*δ*_H_ 7.94 (d, *J* = 8.9 Hz, H-6′), 6.45 (dd, *J* = 8.9, 2.4 Hz, H-5′), 6.35 (d, *J* = 2.4 Hz, H-3′), 7.43 (d, *J* = 8.5 Hz, H-6), 6.47 (d, *J* = 2.4 Hz, H-3), and 6.42 (dd, *J* = 8.5, 2.4 Hz, H-5)), four olefinic protons (*δ*_H_ 7.27 (d, *J* = 14.5 Hz, H-*α*), 7.68 (dd, *J* = 14.5, 11.3 Hz, H-*β*), 7.17 (dd, *J* = 15.6, 11.3 Hz, H-*γ*), and 7.35 (d, *J* = 15.6 Hz, H-*δ*)), and a distinctive signal of a hydrogen-bonded hydroxy group (*δ*_H_ 13.68). The ^13^C NMR data (Table 1) exhibited resonances for a keto-carbonyl (*δ*_C_ 192.8), twelve aromatic carbons (*δ*_C_ 103.7–167.6), and four olefinic carbons (*δ*_C_ 122.1 (C-*α*), 147.3 (C-*β*), 125.0 (C-*γ*), and 139.5 (C-*δ*)). These were characteristic of those reported for (2*E*,4*E*)-1,5-diarylpenta-2,4-dien-1-one [10,11]. The HMBC correlations (Figure 2) from OH-2′ to C-1′ and C-3′, from H-3′ to C-1′ and C-4′, from H-5′ to C-1′, from H-6′ to C-2′ and C-4′, from H-3 to C-1, C-2, and C-4, from H-5 to C-1, and from H-6 to C-2 and C-4 indicated that four hydroxy groups were located at C-2′, C-4′, C-2, and C-4. Moreover, the HMBC correlations from H-6′, H-*α*, and H-*β* to C=O, from H-*α* to C-*γ*, from H-*β* to C-*δ*, from H-*γ* to C-*α*, from H-*γ* to C-*β* and C-1, and from H-*δ* to C-*β* and C-6 suggested the presence of the *α*,*β*,*γ*,*δ*-unsaturated carbonyl moiety in **1**. The NOESY correlations between H-5′ and H-6′, H-6′ and H-*α*, H-*β* and H-*δ*, H-*γ* and H-6, and H-6 and H-5 indicated the relative configuration of **1** as shown in Figure 2. Thus, the structure of semedienone (**1**) was concluded as 2*E*,4*E*-1,5-bis(2,4-dihydroxyphenyl)penta-2,4-dien-1-one.

Compound **2** showed the HRESIMS ion at *m/z* 347.0902 [M + Na]^+^ (calcd. for C_19_H_16_O_5_Na, 347.0895). Its IR spectrum showed absorption bands for hydroxy (3305 cm^−1^) and carbonyl (1650 cm^−1^) groups. The ^1^H and ^13^C NMR spectra of **2** (Table 1) showed signals for two 1,2,4-trisubstituted aromatic rings, which resembled those of **1**. Compound **2** showed the presence of six olefinic protons (*δ*_H_ 7.26 (d, *J* = 14.5 Hz, H-*α*), 7.61 (dd, *J* = 14.5, 11.5 Hz, H-*β*), 6.62 (dd, *J* = 13.7, 11.5 Hz, H-*γ*), 7.00 (2H, m, H-*δ* and H-*ε*), and 7.13 (d, *J* = 14.6 Hz, H-*ζ*)) in the ^1^H NMR spectrum, and six olefinic carbons (*δ*_C_ 122.8 (C-*α*), 145.8 (C-*β*), 129.4 (C-*γ*), 145.8 (C-*δ*), 126.1 (C-*ε*), 134.3 (C-*ζ*)) in the ^13^C NMR spectrum. These were characteristic of those reported for (2*E*,4*E*,6*E*)-1,7-diarylhepta-2,4,6-trien-1-one [12]. The locations of four hydroxy groups were assigned at C-2′, C-4′, C-2, and C-4 by the observed HMBC correlations (Figure 1). Moreover, the HMBC correlations from H-6′, H-*α*, and H-*β* to C=O, from H-*α* to C-*γ*, from H-*γ* to C-*β*, from H-*δ* to C-*ζ*, from H-*ε* to C-*ζ* and C-1, and from H-*ζ* to C-*δ* and C-*ε* suggested the presence of the *α*,*β*,*γ*,*δ*,*ε*,*ζ*-unsaturated carbonyl moiety in **2**. The relative configuration of **2** was deduced based on the NOESY correlations between H-5′ and H-6′, H-6′ and H-*α*, H-*α* and H-*γ*, H-*β* and H-*δ*, H-*γ* and H-*ε*, H-*ε* and H-6, and H-6 and H-5 (Figure 2). Thus, the structure of semetrienone (**2**) was established as 2*E*,4*E*,6*E*-1,7-bis(2,4-dihydroxyphenyl)hepta-2,4,6-trien-1-one.

### 3.3. Tyrosinase Inhibitory Activity of Isolated Compounds from *S. caudata*

Compounds (**1**–**7**) were tested for their tyrosinase inhibitory activity [13]. Kojic acid, a purported skin-lightening agent, was used as a positive control. 2,4,2′,4′-Tetrahydroxychalcone (**8**), which was synthesized following our previous procedure [14], showed potent activity with an IC_50_ value of 0.016 *μ*M (Table 2). Semedienone (**1**) and semetrienone (**2**) exhibited remarkable inhibitory effect with the IC_50_ values of 0.033 and 0.11 *μ*M, respectively, more potent than that of kojic acid (IC_50_, 44.6 *μ*M). Additionally, compounds **4** and **6** were found to possess tyrosinase inhibitory activity with the IC_50_ values of 2.35 and 27.0 *μ*M, respectively.

The presence of *α*,*β*-unsaturated hydroxycarbonyl groups in cinnamic acid derivatives were found to enhance activity (**2** ≫ **3**). Additionally, the occurrence of a C-3 methoxy group decreased the inhibitory activity (**2** ≫ **5**) [15,16]. Diarylalkanoids with 2,4-disubstituted resorcinol subunit on ring B contributed the most to inhibitory activity [17]. Moreover, the length of the conjugated carbon chain in diarylalkanoids led to a change of activity (**8** > **1** > **2**). This result reaffirmed the (*Z*)-*β*-phenyl-*α*,*β*-unsaturated carbonyl scaffold plays an important role for tyrosinase inhibition [18,19]. In previous reports, diarylpentanoids such as diarylpentadiene-3-one were not significantly inhibiting tyrosinase activity [20], but some analogues showed moderate antimelanogenesis activity [21]. Some cyclic diarylheptanoids were found to have melanogenesis-inhibitory activity [22]. In this regard, semedienone (**1**) and semetrienone (**2**) could be the potent structural templates for developing new skin-lightening agents.

### 3.4. Docking Study of the Active Compounds **1**, **2**, and **8**

Tyrosinase has four possible oxidation states (*deoxy*-, *oxy*-, *met*-, and *deact*-form) [23]. *Met*-tyrosinase, having a hydroxy and the two Cu^2+^ ions in the binding site, is responsible for the oxidation of catechols. In this oxidizing process, *met*-tyrosinase is reduced to *deoxy*-tyrosinase which rapidly binds dioxygen to give *oxy*-tyrosinase form. *Oxy*-tyrosinase, which is the primary form of the enzyme, oxidizes both phenols and catechols to *o*-quinones by the monooxygenase and oxidase mechanisms, respectively. In the active site of *oxy*-tyrosinase, two bound Cu^2+^ ions and the peroxide group play a catalytic oxidation role. Mushroom tyrosinase (EC 1.14.18.1), which was used in the inhibitory assay, plays the same role with respect to *oxy*-tyrosinase form. Thus, in this study, the molecular docking studies of **1**, **2**, and **8**, respectively, with *oxy*-tyrosinase (PDB ID : 1WX2) [24] were carried out to explore their interactions and inhibition mechanisms.

In molecular docking study, the imperfect scoring results (false-positive hits), which may be considered as decoys, can be occurred by predicting incorrect ligand geometries or by applying nonbinding molecules. The active and decoy ligands are similar according to some physicochemical properties (molecular weight, number of rotational bonds, total hydrogen bond donors, total hydrogen bond acceptors, topological polar surface area, and the octanol-water partition coefficient), but decoy was presumed to be inactive against a target. According to Choi et al. [25], kojic acid and hypoxanthine showed the tyrosinase inhibitory constant (*K*_*i*_) values of 13 *μ*M and >1000 *μ*M, respectively [25]. Thus, in this docking study, kojic acid and hypoxanthine was selected as the active inhibitor and the decoy molecule, respectively, to validate our docking protocol.

The docking studies were performed with Molecular Operating Environment 2016 (MOE 2016.0802) suite [26]. The top-ranked pose with the highest negative binding free energy value (*S* value) was selected for further interaction analysis with BIOVIA Discovery Studio Visualizer 2016 [27].

**Figure 3:**
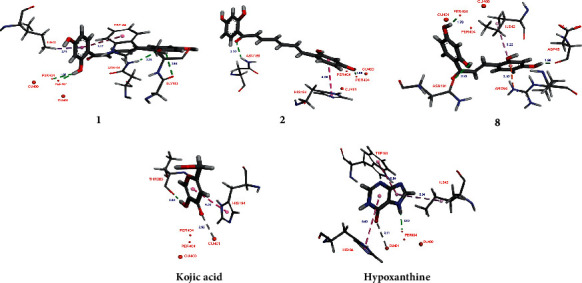
3D docking models of **1**, **2**, **8**, kojic acid, and hypoxanthine with *oxy*-tyrosinase (1WX2).

Compounds **1**, **2**, and **8** showed an H-donor interaction between a hydroxy group and a peroxide bridge PER404, presenting the distances of 1.85, 1.88, and 1.78 Å, respectively, whereas kojic acid showed the interactions with a Cu^2+^ ion, HIS194, and THR203 residues (Figure 3). In the binding pocket, compounds **1** and **8** showed more interactions with targeting residues than those of **2** (Table 3). These analysis results were consistent with their experimental inhibitory activities (**8** > **1** > **2**). The C-2 hydroxy group of **1** exhibited H-bonding interactions with ASN191 and GLY183 residues. Moreover, the aromatic ring A of **1** formed *π-π* T-shaped and *π-σ* interactions with TRP184 and ILE42 residues, respectively. Compound **2** showed an H-acceptor interaction between C=O group and ASN188 residue. In addition, the aromatic ring B of **2** interacted with HIS194 residue via a *π-π* stacking interaction. Thus, the *S* values and these interactions suggested that **1** and **2** showed high binding affinity for *oxy*-tyrosinase than those of kojic acid. Hypoxanthine showed the lower negative *S* value and the longer-distance interactions than that of kojic acid. Apparently, these results could be used to validate the abovementioned docking procedure in this study.

### 3.5. Proposed Biosynthetic Pathways of **1** and **2**

**Figure 4:**
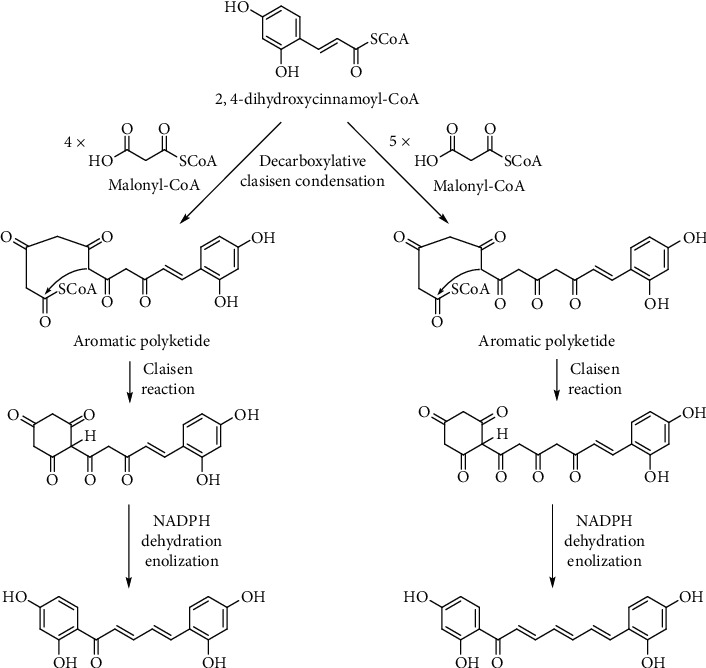
Plausible biosynthetic pathways for diarylalkanoids **1** and **2**.

We have proposed plausible biogenetic pathways for two new diarylalkanoids (**1** and **2**) (Figure 4) *via* the shikimate and acetate pathways [28]. *α*-Ketoglutarate-dependent hydroxylase is responsible for the C-2 hydroxylation of *p*-coumaroyl-CoA to give 2,4-dihydroxycinnamoyl-CoA [29]. It is condensed with four or five malonyl-CoA moieties to afford the corresponding polyketides, which undergo the intramolecular ring closure *via* Claisen reaction. After that, reduction, dehydration, and enolization must occur to give rise to **1** and **2**.

## 4. Conclusions

From the CHCl_3_-soluble extract of the stems of *S. caudata*, two new diarylalkanoids were isolated together with five known compounds. Compounds **1** and **2** were found to possess potent tyrosinase inhibitory activity with the IC_50_ values of 0.033 and 0.11 *μ*M, respectively. Binding interaction analyses between the *oxy*-tyrosinase active site and the active compounds (**1** and **2**) have been performed. Plausible biogenetic pathways for formation of two new diarylalkanoids (**1** and **2**) were also proposed.

## Figures and Tables

**Table 1:** ^1^H (500 MHz) and^13^C (125 MHz) NMR data (acetone-*d*_6_) for compounds **1** and **2**.

Position	1		2	
	*δ*_C_, type C	*δ*_H_ (*J*, Hz)	*δ*_C_, type C	*δ*_H_ (*J*, Hz)
1′	114.5, C		114.9, C	
2′	167.6, C		167.4, C	
3′	103.8, CH	6.35, d (2.4)	103.8, CH	6.35, d (2.4)
4′	165.5, C		165.9, C	
5′	108.7, CH	6.45, dd (8.9, 2.4)	108.8, CH	6.45, dd (8.9, 2.4)
6′	132.9, CH	7.94, d (8.9)	132.6, CH	7.92, d (8.9)
C=O	192.8, C		192.6, C	
*α*	122.1, CH	7.27, d (14.5)	122.8, CH	7.26, d (14.5)
*β*	147.3, CH	7.68, dd (14.5, 11.3)	145.8, CH	7.61, dd (14.5, 11.5)
*γ*	125.0, CH	7.17, dd (15.6, 11.3)	129.4, CH	6.62, dd (13.7, 11.5)
*δ*	139.5, CH	7.35, d (15.6)	145.8, CH	7.00, m
*ε*			126.1, CH	7.00, m
*ζ*			134.3, CH	7.13, d (14.6)
1	116.5, C		116.6, C	
2	158.5, C		157.8, C	
3	103.7, CH	6.47, d (2.4)	103.7, CH	6.45, d (2.4)
4	161.0, C		161.0, C	
5	108.9, CH	6.42, dd (8.5, 2.4)	108.8, CH	6.39, dd (8.5, 2.4)
6	130.2, CH	7.43, d (8.5)	129.3, CH	7.39, d (8.5)
OH-2′		13.68, s		13.64, s

**Table 2:** Tyrosinase inhibitory activities of the isolated compounds **1**–**8**.

Compound	IC_50_ (*μ*M)
1	0.033
2	0.11
3	>100
4	2.35
5	>100
6	27.0
7	>100
8	0.016
Kojic acid ^*a*^	44.6

^*a*^Positive control.

**Table 3:** Docking results of **1**, **2**, **8**, kojic acid, and hypoxanthine with *oxy*-tyrosinase.

Compound	*oxy*-tyrosinase (1WX2)			
	*S* values	Interactions	Targeting residues	Distance (Å)
**1**	–5.75	H-donor	PER404	1.85
			ASN191	2.25
			GLY183	2.85
		*π*-*σ*	ILE42	2.75
		*π*-*π*	TRP184	5.17
**2**	–6.37	H-donor	PER404	1.88
		H-acceptor	ASN188	2.30
		*π*-*π*	HIS194	4.30
**8**	–5.54	H-donor	PER404	1.78
			ASP45	1.96
			ASN191	2.83
		*π*-alkyl	ILE42	5.22
		*π*-cation	ARG55	3.10
Kojic acid ^*a*^	–4.50	H-donor	THR203	2.04
		Metal-acceptor	CU401	2.92
		*π*-*π*	HIS194	4.30
Hypoxanthine ^*b*^	–4.34	H-donor	PER404	2.05
		Metal-acceptor	CU401	3.11
		*π*-*π*	HIS194	5.40
			TRP184	5.04
		*π*-alkyl	ILE42	5.04

^*a*^Positive control.  ^*b*^Decoy molecule.

## Data Availability

The data used to support the findings of this study are included within the article.

## Supplementary Materials

Supplementary Materials
^1^H and ^13^C NMR spectra of two new compounds, semedienone (1) and semetrienone (2).
