# Supplementary material for: Diarylalkanoids as Potent Tyrosinase Inhibitors from the Stems of Semecarpus caudata
Source: Evid Based Complement Alternat Med. 2021 Jan 4;2021:8872920. doi: 10.1155/2021/8872920 (PMC7801053; doi:10.1155/2021/8872920)
Supplement: Supplementary Materials — 1H and 13C NMR spectra of two new compounds, semedienone (1) and semetrienone (2). [file 8872920.f1.docx]

***Supplementary data***

# **Diarylalkanoids as Potent Tyrosinase Inhibitors from the Stems of *Semecarpus caudata***

Phu H. Dang, Tho H. Le, Truong N. V. Do, Hai X. Nguyen, Mai T. T. Nguyen, and Nhan T. Nguyen

## **Abstract**

From a CHCl_3_-soluble extract of the stems of *Semecarpus caudata* (Anacardiaceae), two new diarylalkanoids, semedienone (**1**) and semetrienone (**2**), were isolated. Their structures were elucidated based on NMR spectroscopic data interpretation. These compounds possess strong tyrosinase inhibitory activity with the IC_50_ values of 0.033 and 0.11 *µ*M, respectively. Docking studies of **1** and **2** with *oxy*-tyrosinase were carried out to analyze their interactions. Accordingly, semedienone (**1**) showed good interactions with the peroxide group and amino acid residues. The biosynthesis of the isolated diarylalkanoids was proposed.

***Keywords:*** *Semecarpus caudata*; Diarylalkanoids; Tyrosinase inhibitory; Molecular docking.

**Figure S1.** ^1^H NMR spectrum of **1** (acetone-*d*_6_, 500 MHz)

**Figure S2.** ^13^C NMR spectrum of **1** (acetone-*d*_6_, 125 MHz)

**Figure S3.** HSQC NMR spectrum of **1**

**Figure S4.** HMBC NMR spectrum of **1**

**Figure S5.** NOESY NMR spectrum of **1**

**Figure S6.** HRESIMS of **1**


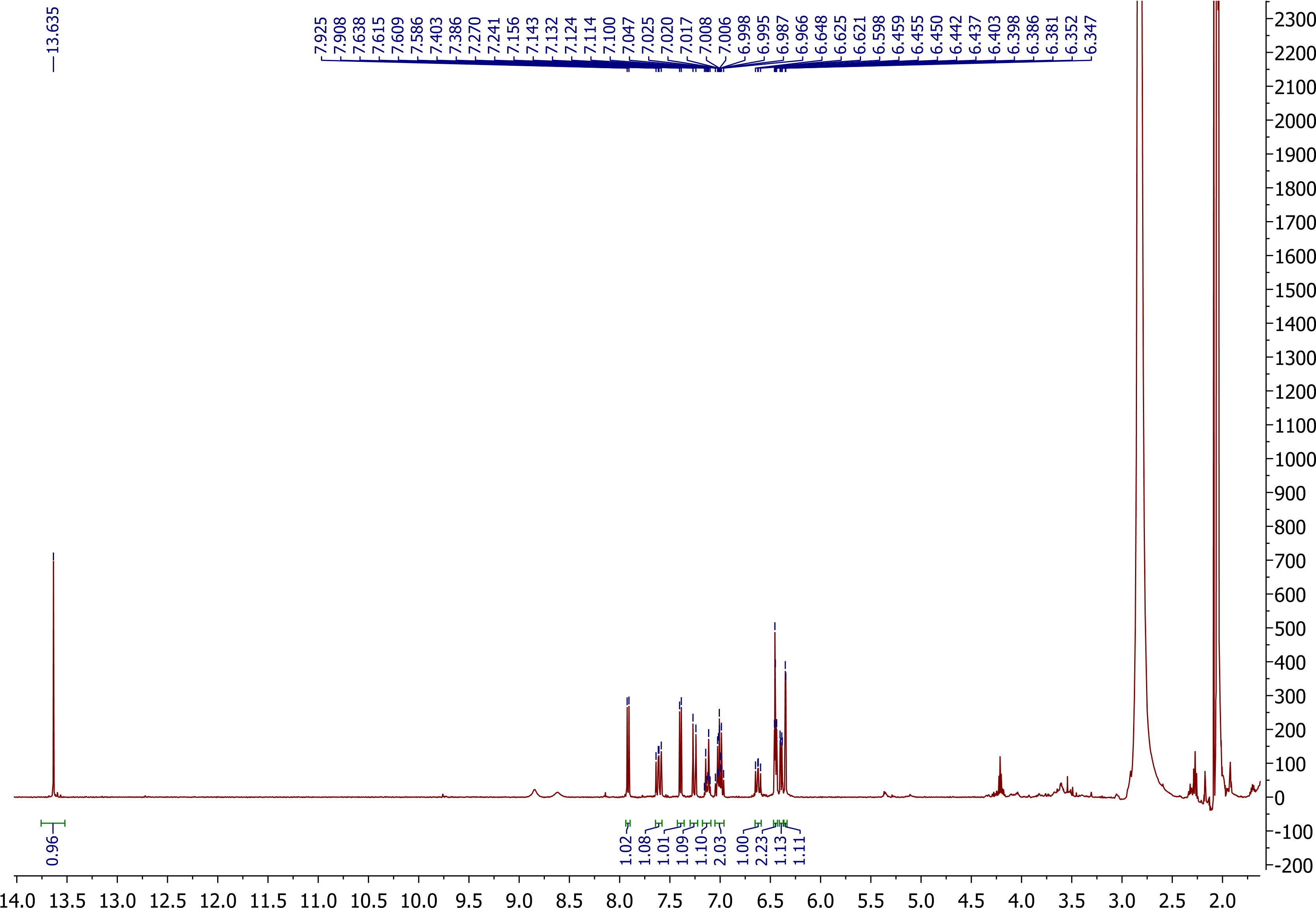


**Figure S7.** ^1^H NMR spectrum of **2** (acetone-*d*_6_, 500 MHz)

**Figure S8.** ^13^C NMR spectrum of **2** (acetone-*d*_6_, 125 MHz)


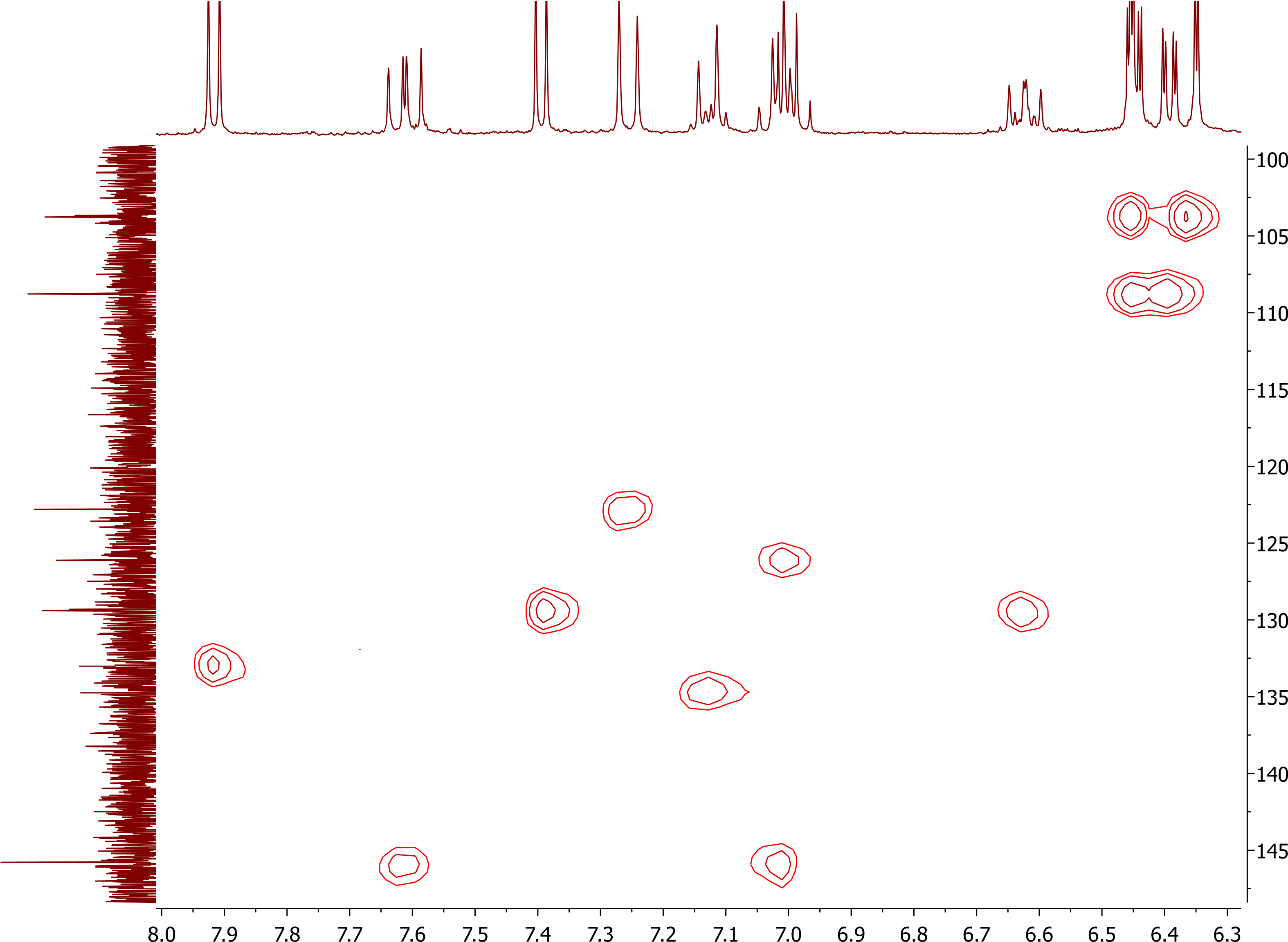


**Figure S9.** HSQC NMR spectrum of **2**


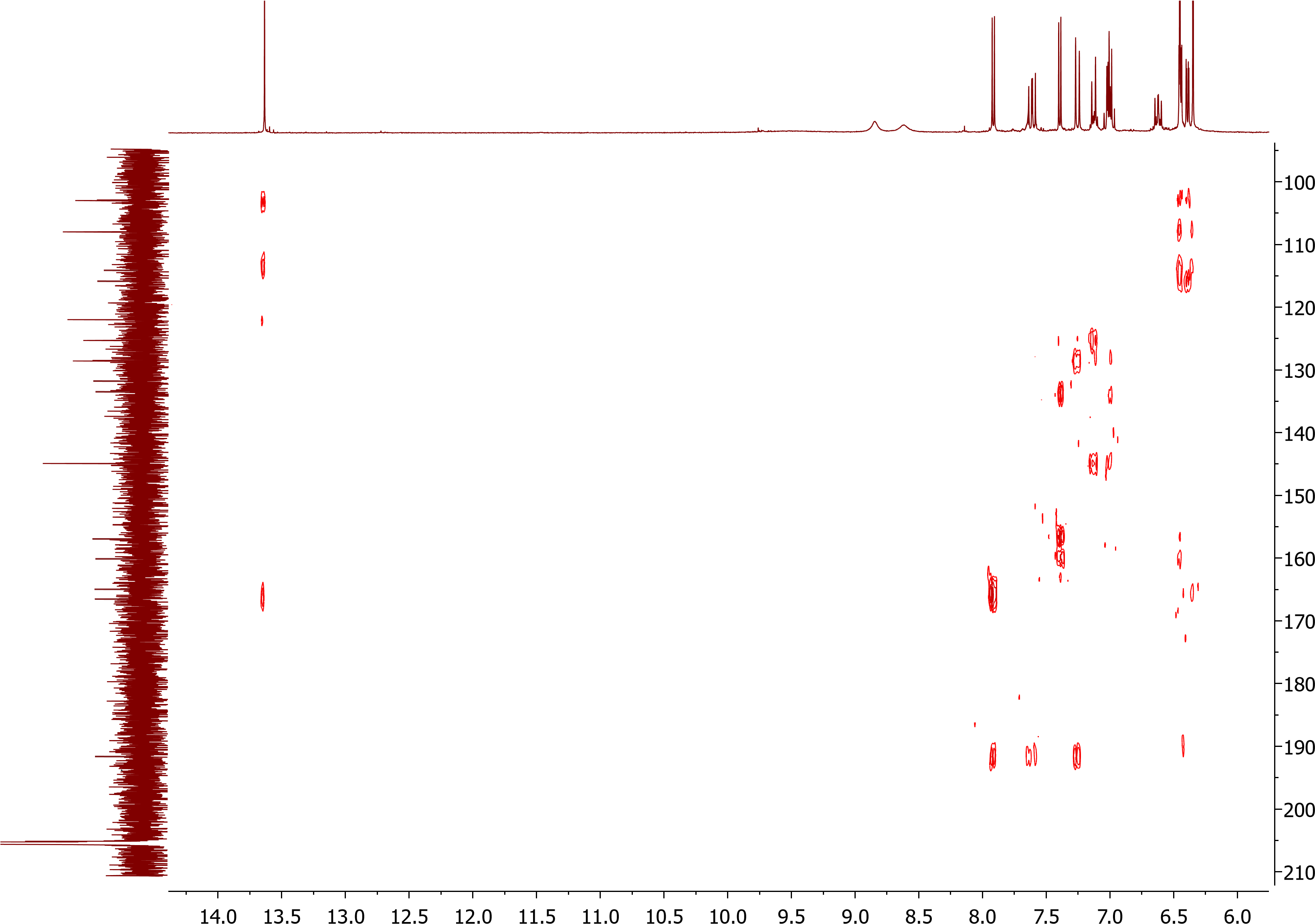


**Figure S10.** HMBC NMR spectrum of **2**


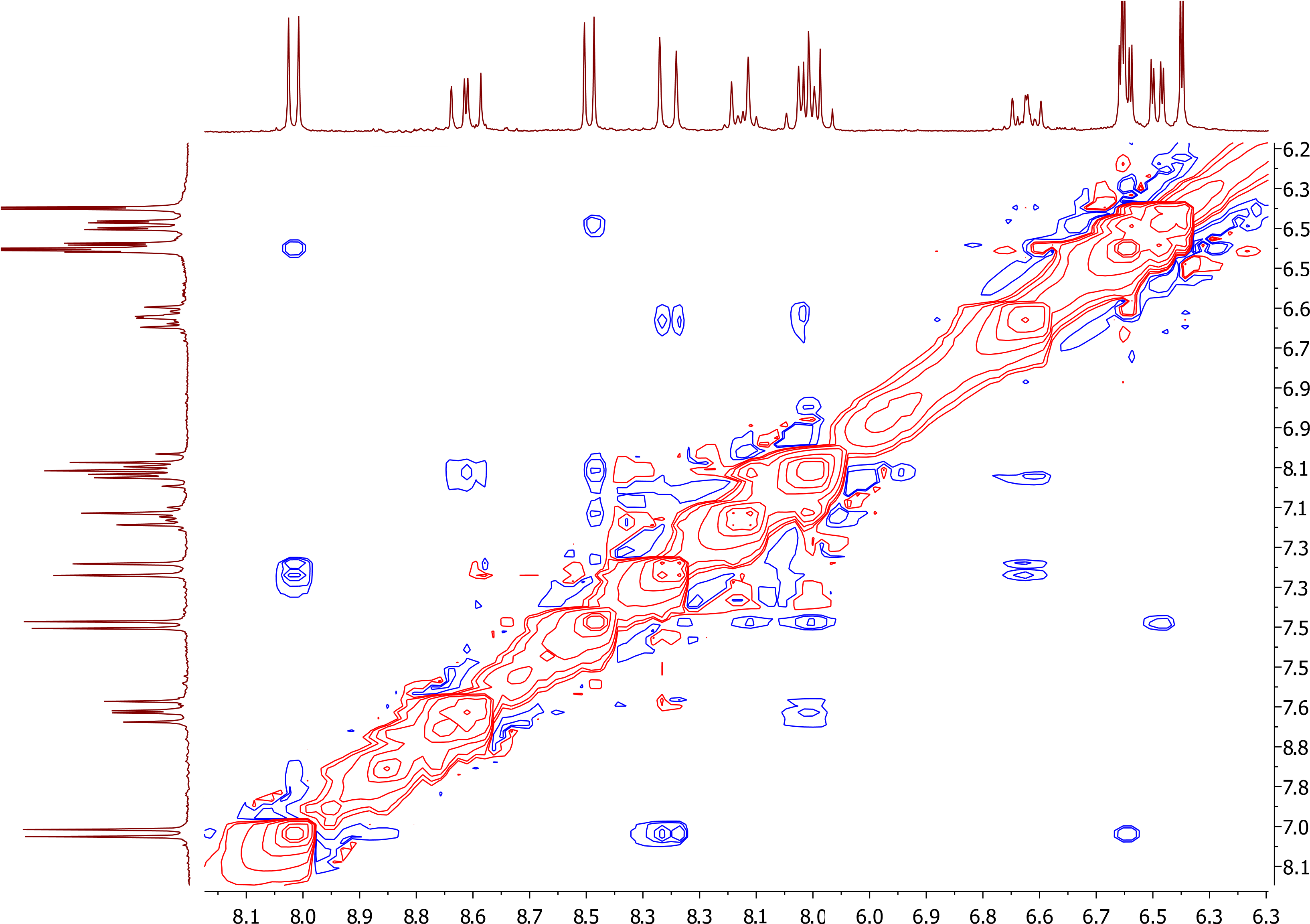


**Figure S11.** NOESY NMR spectrum of **2**

**Figure S12.** HRESIMS of **2**

**
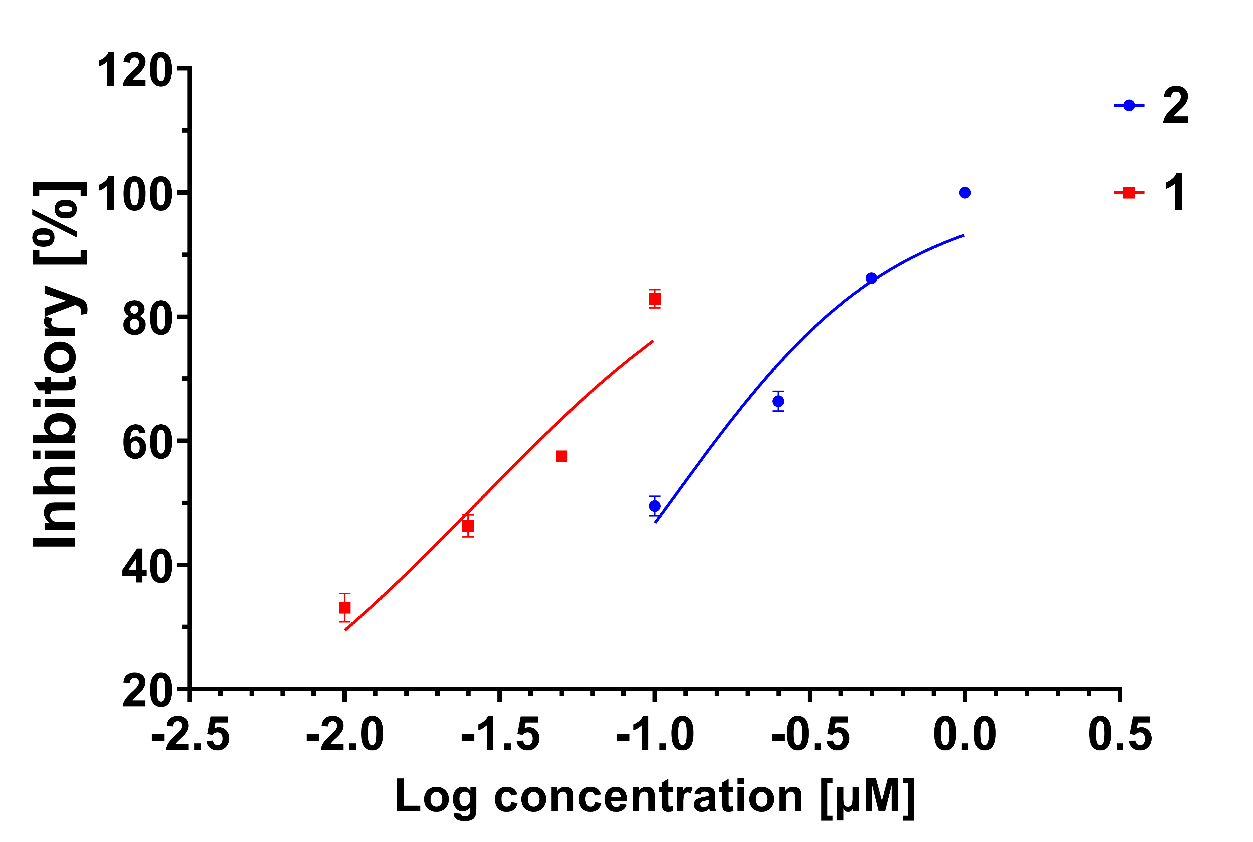
**

**Figure S13.** Dose-dependent tyrosinase inhibitory activity of **1** and **2**
